# A Single Amino Acid Change to Taq DNA Polymerase Enables Faster PCR, Reverse Transcription and Strand-Displacement

**DOI:** 10.3389/fbioe.2020.553474

**Published:** 2021-01-14

**Authors:** Wayne M. Barnes, Zhian Zhang, Milko B. Kermekchiev

**Affiliations:** ^1^Department of Biochemistry and Molecular Biophysics, Washington University School of Medicine, St. Louis, MO, United States; ^2^DNA Polymerase Technology, Inc., St. Louis, MO, United States

**Keywords:** diagnostics, polymerase chain reaction with reverse transcription, Taq DNA polymerase, RNA detection, RT-LAMP, RT-PCR

## Abstract

A change of an aspartic acid to asparagine of Taq (*Thermus aquaticus*) DNA polymerase is a gain of function mutation that supports faster PCR: the extension times for PCR amplification can be 2–3 times shorter. Surprising results from negative controls led to the discovery of strand-displacement ability and reverse transcriptase activity of Taq D732N DNA polymerase. We demonstrate that the mutant enzyme can, by itself, catalyze RT-PCR, and RT-LAMP assays. Residue 732 is on the surface of the enzyme, not near the active site.

## Introduction

Real-time quantitative polymerase chain reaction (qPCR), catalyzed by *Thermus aquaticus* DNA polymerase (Taq pol) can be used for food safety tests to identify the presence of harmful bacteria in food or feed. When used for this purpose, some compounds in food, such as chocolate or black pepper, are inhibitors of the DNA polymerase, and require extreme dilution and/or extensive purification to allow effective qPCR (Rossen et al., 1992; Sovová et al., 2017). We conducted a search for variants of Taq DNA polymerase with increased tolerance to inhibitors in food, using an aqueous extract of black pepper at a level that greatly reduces the efficiency of qPCR. From a library of 4000 mutagenized clones of Taq DNA polymerase, we describe one of the enzymes that outperformed in qPCR, compared to the rest of the mutagenized library (Supplementary Figure 1). We characterized the purified mutant enzyme and its N-terminally deleted version (Klentaq1), compared to the wild-type versions, and found surprising features.

We discovered that *Thermus aquaticus* DNA polymerase is only one amino acid change away from being an efficient reverse transcriptase and being capable of strand-displacement. These two assay functions are commonly achieved by the use of less thermostable DNA polymerases similar in sequence and structure, or by inclusion of Mn++ ion. These gains in function resulted from a change of aspartic acid to asparagine at position 732, a residue on the surface of Taq pol, some 30 Å from the active site. The discovery process entailed positive results from negative controls, to wit: For reverse transcriptase (RT) activity, Taq pol by itself is the usual no-RT control for RT-PCR, yet Taq D732N by itself produced RT-PCR products, unless the RNA template was first treated with RNase I. For strand-displacement, the 5′-flap endonuclease/exonuclease of Taq pol was expected to be the reason that full-length Taq pol cannot perform LAMP, which requires priming on double-stranded DNA and strand-displacement without concomitant degradation of the displaced strand. For this reason, our initial strand-displacement experiments with D732N employed an N-terminal deletion of the 5′-exonuclease (Klentaq1), and full-length Taq D732N was the negative control, but was not negative. We describe conditions under which, with the new functions, Taq D732N can carry out RT-PCR, LAMP, and RT-LAMP. We also show that, compared to wild-type Taq pol, and by amplifying various lengths of human DNA, plain PCR can be performed with extension times that are 2–3 times shorter per PCR cycle.

## Materials and Methods

Codon numbers (amino acid numbers) throughout are those for the wild-type (full-length Taq) *Thermus aquaticus* DNA polymerase gene coding for 832 amino acids (Lawyer et al., 1989), NCBI GenBank Accession no. J04639.1. Klentaq1 is an N-terminal deletion of 278 amino acids of Taq DNA polymerase, which removes the domain for 5′-flap endonuclease and improves thermostability (Barnes, 1995). Table 1 shows the sequence context of the mutations studied here, using the single letter code for amino acids.

**TABLE 1:** Mutated amino acid positions.

Mutation of Taq pol	Amino acid context	Codon context
D732N	PDL to PNL	CCAaACCTA
D119N	ADD to AND	GCGaACGAC
D119A	ADD to AAD	GCGgcgGAC
D142A	ADK to AAK	GCCGcgAAA
Klentaq1	starts as MGLLHEF	GGCAGC
		CTCCTCCACGAGTTC

### Agarose Gels

Unless otherwise specified, the Lonza FlashGel webcam system was used to observe 1.2% agarose gels while running and to make photographs.

#### Expression and Purification of DNA Polymerases

The ORF (open reading frame) for wild-type *Thermus aquaticus* DNA polymerase was mutagenized by error-prone PCR as described (Cadwell and Joyce, 1992; Kermekchiev et al., 2003) and cloned into plasmid pUC19 using standard methods, to make a mutagenized library. The host bacterial strain (designated *E. coli*) that can stably abide and express full-length Taq pol plasmid was a gift from Clontech without genotype information. The ORF for Klentaq1 D732N was assembled from 400 bp pieces (IDT) consisting of codons optimized for *E. coli* expression and cloned into a custom pET-like vector pWB978, resulting in expression plasmid pKT732N. The *E. coli* host strain WB532 was derived from WB466.15 and carried a mutant T7 RNA polymerase gene between Z and Y of the lac operon as described (Barnes, 2015). The variety of Taq and Klentaq enzymes, including the ones wild-type at codon 732, were expressed and purified as described (Kermekchiev et al., 2003). The final purification step was adsorption to heparin sepharose (Sigma) and elution with storage buffer: 50% glycerol v/v containing 222 mM ammonium sulfate, 20 mM Tris pH 7.9, 0.2% Thesit, and 10 mM 2-mercaptoethanol. Dilutions were made in this same storage buffer. Crucially for the determination of enzyme concentration, the stabilizing non-ionic detergent used during purification and storage was Thesit (Sigma), which has very low OD280 compared to several other detergents in common use (Triton X100, Tween 20, NP40, IGEPAL). Purified DNA polymerases were used from a stock concentration at OD280 = 0.8 or 1.0 or 0.16, as indicated (0.8, 1, or 0.16 mg/ml). 1 μl (1 μg) was used per 25 μl reaction volume for LAMP experiments, and 0.16 μg was used for PCR, per 25 μl reaction volume, unless indicated otherwise. Bst pol was supplied as Large Fragment by New England Biolabs or as Manta from Enzymatics, at an estimated 0.2 and 1 mg/ml, respectively. We have found that deletions and mutations of Taq DNA polymerase have various levels of Kornberg units per ug. For best reproducibility, we use OD 280 (extinction coefficient 1 per mg/ml) for quantitation.

#### DNA Sequencing

The Taq or Klentaq1 ORF and flanking DNA was PCR-amplified from plasmid mini preps from purified single colonies and sent to GENEWIZ for Sanger DNA sequence confirmation. Sequence data chromatograms were analyzed using software phred, phrap, and consed (Ewing and Green, 1998; Gordon, 2003). As the plasmid encoding TaqD732N was modified to have additional mutations at codon 119 and 142, confirmations of the sequence over the full-length of the TaqD732N ORF resulted in resequencing at least six times.

Templates and primers for test amplifications are listed in Table 2. Primers were supplied by IDT. TEN (10 mM Tris–HCl pH 8, 0.1 mM EDTA, 10 mM NaCl) was used to dilute and store all primers.

**TABLE 2:** Templates and primers.

Experiment	Template	Left primer(s)	Right primer(s)
Crude lysate PCR screen	None added, except as the crude bacterial extract/enzyme. PCR target was rDNA.	DG74: AGGAG GTGATCCAACCGCA (Teng et al., 2004)	RW01; AACTG GAGGAAGGTGGGGAT
PCR speed test	Human DNA (Novagen) 3.7 ng per 40 μl reaction; CCR5 gene was targeted.	16.136 CCTGACAATC GATAGGTACC TGGCTGT	16.125′ CTTCT CATTT CGACA CCGAA GCAGA (270 bp gap);17.70′ CGTTT CTGAA CTTCT CCCCG ACAAA GGC (502 bp gap);17.71′ TCTGT TCAGA TCACT AAACT CAAGA ATC (1000 bp gap)
LAMP assay for DNA	Phage lambda DNA (New England Biolabs), 1 ng per 25 μl reaction.	14.163 F3 lambda GTAAA AACAC CTCAC GAGTT;14.165 FIP lambda: TCCTA CGGTC AAGAG AAGCAATAAA (-) CACCT AAGTT CTCAC CGAAT	14.164 B3 lambda TTTAC GAACA TTAAG CGACTT;14.166 BIP lambda: CTTTCCACATGC AGGAT TTTGG (-) ATG CACGC AATGG TGTAG
MS2 RT-LAMP	MS2 RNA (Roche) as 1 μl of 2–3 ng/μl in 0.1X reaction buffer. Primer set suggested by Chander et al. (2014).	15.36 MS2F3 TGTCA TGGGA TCCGG ATGTT; 15.38 MS2FL CAGAG AGGAG GTTGCCAA; 15.40 + Mly MS2FIP GCCC AAAC AACGA CGATC GGTA gagtc AAAC CAGCA GTAGCCT;	15.37 MS2B3 CAATA GAGCC GCTCT CAGAG;15.39 MS2BL TGCAG GATGC AGCGCCTTA; 15.41 + Mly MS2BIP GCACGTTC TCCAACG GTGCT gagtc GGTTG TTGT TCAGC GAACT.
MS2 RT PCR	Same as above, but only 5 pg per reaction.	MS2-P-FOR: GTATC TTGAA CCCAC TAGGT ATAG;MS2-K-FOR: AGTTC GGTTG GTTAC CACTAAT	MS2-P-REV: CGACG AGAAC GAACTGAGTAA;MS2-K-REV: AAGGT ATGGA CCATC GAGAAAG

### Specific Protocols

#### PEG + SDS Precipitation

This DNA and RNA product purification method consisted of the addition of 6–8 μl of 0.5% blue dextran [treated as 1% with DEPC (diethyl pyrocarbonate 0.3% final), 2.5% SDS], 1/2 volume of PEG-NaCl (30% polyethylene glycol 3350, 1.5 M NaCl) to a DNA sample of 100–250 μl, followed by centrifugation for 15–30 min at 10,000 rpm. The visible blue pellet was rinsed with 75% ethanol, dried in air for 10–30 min and resuspended in TEN for DNA, or DEPC-treated water for RNA.

#### PCR Speed Tests

Each master mix for 9.2 reactions (368 μl) contained 1.1 μl of either wild-type Taq pol or TaqD732N from a stock at OD280 = 0.16. Final dNTP (New England Biolabs) were at 200 μM each, and the primers were at 400 nM. Reaction buffer was 50 mM Tris–HCl pH 8.3 at 25°C, 2.9 mM MgCl2, 8 mM ammonium sulfate, 0.025% Brij-58. The thermal cycler (SimpliAmp) was set to an initial 90 s 94°C to denature the human DNA template, then 34 cycles of 1 s 94°C, X s 65°C, then a final 1 min at 65°C, where X was varied from a minimum setting of 1 s. A setting of 1 s entails a few seconds near the target temperature, due to machine hysteresis. 1.5 μl of PCR product was analyzed on 1.2% agarose gels.

#### Half-Dumbbell Construction

A DNA “handle” (Figure 2A) was amplified from phage lambda DNA by PCR using primers 13.16 (GGCAT TGTTT GGTAG GTGAG AGATCT) and 13.17′ (ACAAA TGACA AGAGT CTGGT TCAGA AGATA), precipitated with PEG + SDS, and digested with restriction enzymes *Hin*dIII and *Bgl*II at its opposite ends. A stem loop consisting of self-annealed, 5′-phosphorylated DNA 11.164 (5P-agct C TGT CTC TTA TAC ACA TCT aata GTTTAACTTTAAGAAGGAGATATA aata AGATGTGTATAAGAGACAG) was added using T3 ligase (Enzymatics) in T4 ligase buffer (NEB) supplemented with 150 mM NaCl. This product was precipitated with PEG + SDS and resuspended at 10 μg per ml in 1x CutSmart buffer (NEB). To each 1.5 μg of DNA was added 1 μl T7 exonuclease (NEB) and incubated on ice as described Barnes (2016) for 40 min to allow removal of ca. 50 bases from the 5′-*Bgl*II end, followed by heat denaturation of the exonuclease at 75°C for 15 min. In a buffer of TEN + 200 mM NaCl, primer 13.16 (GGCATTGTTTGGTAGGTGAGAGATCT) was annealed to replace some of the bases removed by the T7 exonuclease, with a gap in “front” of it at its 3′-end. The pellet from PEG + SDS precipitation was rinsed with 75% ethanol containing 200 mM NaCl, and resuspended in TEN + 150 mM NaCl.

#### RT-LAMP MS2 RNA Primer Mixture

The six primer sequences were suggested by Lucigen (Chander et al., 2014). An *Mly*I restriction site was inserted into the FL and BL primers, but has not yet been used. The mixture contained 120 μl of 1.05x RT LAMP reaction buffer (see below), 20 μl of F3 and B3 from 10 μM in each stock, and 20 μl of Fip, Bip, FL, and BL from stock 40 μM in each (i.e., ratios 1:4:4), and 1 μl of Antarctic UDG (New England Biolabs). For real time data gathering, 30 μl of 20X Eva Green dye (Biotium) was included. In use, 4 μl of this primer mix (5 μl if Eva Green was used as indicator) was added to the caps of each reaction tube (containing 21–22 μl of each otherwise-complete reaction volume) as a hanging drop hot start (see below).

#### RT-LAMP

This protocol includes several features designed to prevent carry-over of amplified product DNA. Primers for RT-LAMP were aliquoted at a naive off-site location, and dispensed with a pipettor that was not used for gel analysis. All other components of the RT-LAMP reactions were treated with DNase I during reaction setup as described below.

Reactions used some seven times less dNTP and half as much primer compared to many published LAMP reactions (Tomita et al., 2008; Tanner et al., 2012; Chander et al., 2014). KLA PCR buffer (1X = 50 mM Tris–HCl pH 8.55, 2.9 mM MgCl2, 0.025% Brij-58, 8 mM ammonium sulfate) was supplemented with final 0.75 M betaine, 4 mM DTT, 0.1 mM CaCl_2_, and 200 μM each of 5 dNTP (A,G,C,T,U) to make **RT LAMP reaction buffer**. The DTT was included to allow for efficient thermal inactivation of DNase I and *E. coli* RNase I (if used) and the CaCl_2_ was included as cofactor for the DNase I treatment. A fifth triphosphate, dUTP, was included at equimolar concentration to allow the potential use of uracil glycosylase (UDG) to ensure removal of contaminating product from previous amplifications, but UDG was used here only in the primer mix (see above). At time of LAMP reaction setup, the RT LAMP reaction buffer at 1.05X and lacking enzyme and nucleic acid, was supplemented with 1/100 volume of DNase stock (New England Biolabs; final 10 μg/ml DNase) in order to eliminate carry-over DNA contamination. DNA polymerase to be tested was included at a final 1 μg per reaction, or as indicated in the Figures. MS2 RNA (1 μl, 2.5 ng) in 0.1X reaction buffer (or buffer lacking RNA) was dispensed to empty reaction tubes, followed by 21 μl of the DNase-containing RT LAMP reaction buffer at 1.05X and incubated at room temperature for 5–10 min. 5 μl of primer mixture were dispensed to the inside of each test tube cap, for a “hanging drop hot start.” After a further 10 min at room temperature (transport to distant PCR machine lab) reactions were allowed to equilibrate in the hot block of a thermal cycler at 73°C for 30–60 s to inactivate the DNase I in the reaction mix and the UDG enzyme in the primer mix.

##### Hanging drop hot start

As the cycler program switched to 68°C, the cycler was paused. Within a minute, each tube or 8-mer of tubes was given a brisk downward shake to add the primers to the reaction, another downward shake, and returned to 68°C for 50–120 min or indicated time periods, with no temperature cycling. If electrophoresis was used, 1.5–1.7 μl from 50 min reactions was loaded onto 1.2% agarose gels. Real-time LAMP was conducted in a MiniOpticon (Bio-Rad) cycler, and raw fluorescence from EvaGreen (Biotium) was displayed with Bio-Rad CFX Manager software.

##### DNase I treatment caveat

If the described 30–60 s heat treatment was followed by cooler temperatures, such as ice, the DNase retained enough activity to destroy primers (data not shown). Evidently the DNase was inactive, but not completely denatured, only if kept at high temperatures for longer. Our DNase conditions are otherwise very similar to those previously described for master mix treatment followed by DNase inactivation at 80°C (Silkie et al., 2008).

RNase I treatment, when used to demonstrate the requirement for RNA template, was minimal, and carried out in 0.1-strength RT-LAMP reaction buffer, at 2 or 3 ng MS2 RNA per ul in a volume of 25 μl, with 0.2 units of *E. coli* RNase I (Epicentre). Incubation was at ambient temperature for 10 min, then 1 μl of the MS2 RNA (with or without the RNase I) was aliquoted to reaction tubes, followed by addition of 21 μl RT-LAMP reaction mix with DNase I. The presence of the 1 μl droplet of RNA was visually confirmed for each reaction before addition of reaction mix.

#### RT-PCR

A pre-incubation step of 30 min at 68°C was employed to allow the reverse transcription, followed immediately by programmed PCR amplification. RT step: 2 min 75°, 2 min 56°, 30 min 68°. PCR cycling: 2 min 95° followed by 35 cycles of 25 s 95°, 40 s 56°, 90 s 70°). Reaction buffer was (1X) 50 mM Tris–HCl, pH 9.1, 16 mM AmSO_4_, 2.5 mM MgCl_2_, 0.025% Brij-58, 200 μM each dNTP.

#### One-Step Reverse Transcription to Compare Wild-Type Taq Pol to Taq D732N

DNA template, for use by T7 RNA polymerase, was prepared by PCR using primer 13.305 (T7 promoter) GAAAT TAATACGACT CACTA TAGGG) and primer 17.1′ (CCCTG AGCTG GATCC AGCAG GTAGG) and arbitrary plasmid pWB972FF to make a 415 bp DNA having a pT7 promoter.

RNA was transcribed from the 415 bp PCR product using the New England Biolabs HiScribe T7 kit, treated with DNase I and precipitated with 10% PEG. The predicted RNA template sequence of 393 nucleotides is shown in Supplementary Figure 7A, along with the positions of four complementary primers used to prime reverse transcription by wild-type Taq pol and mutant Taq D732N (A111).

The reaction buffer was the same as for RT-PCR, except that dATP concentration was reduced to 80 μM when labeling with α-^32^P-dATP, and final 1 M betaine was included. Each 16 μl contained final 1 pmole of RNA and 2.5 pmole of primer, and 40 ng (0.43 pmole) of either wild-type Taq or Taq D732N, both home-made. Importantly and essentially, the RNA and water portions of the master mix were heated together at 75–80°C for 5 min before cooling on ice and combining with all non-primer ingredients. 6.75 μl of reactions lacking primer were started with 1.25 pmoles primer as 1.25 μl and incubated at 68°C for 5, 10, 15, 20, or 30 min as indicated in Supplementary Figures 7B, 8. The reactions were halted by moving to wet ice for 1 min, and further stopped with excess EDTA (2 μl of 15 mM). After spin vacuum to reduce the volume to less than 2 μl, 6 μl of formamide were added. After heating to 94°C for 1 min, approximately 2 μl were loaded onto a 7 M urea, 10% acrylamide sequencing gel of thickness 0.4 mm. Phosphor screen (Bio-Rad) exposures to fixed (by 10% acetic acid) gels were for 15–18 h.

Size standards included in Supplementary Figure 7B were prepared by T7 exonuclease digestion of 32P-labeled PCR products anchored on one side by primer 13.305 having 5 phosphorothioate linkages (G^∗^A^∗^A^∗^A^∗^T^∗^ TAATACGACT CACTATAGGG). This did not produce clean single bands of single-stranded DNA. Size comparisons are approximate, since these size standards are not the identical DNA sequence, and denaturation on the gel (10% acrylamide, 7 M urea) is not complete. No trap DNA was included, so multiple enzyme/DNA binding events are possible, as for the RT step of a typical RT-PCR.

## Results

### Screen for Taq Pol Mutations Using Crude Lysates

We used total *E. coli* crude extract both as a source of enzyme and as the source of PCR template to screen circa 4,000 mutagenized clones that expressed Taq DNA polymerase from a plasmid (vector pUC19). We seeded 100 μl cultures with 3–10 clones each, and induced expression in 96-well plates (1 mM IPTG, 14–16 h with 150 rpm shaking). We added PCR primers for ribosomal DNA, reaction buffer, dNTPs and SYBR Green as 35 μl master mix, to 3–5 μl of crude enzyme extract in 96-well clear PCR plates (Bio-Rad), followed by immediate temperature cycling (50 cycles of 30 s 94, 40 s 54, final 1 min 70) Although an inhibitor (cracked pepper extract) was included in the screen, purified enzyme did not demonstrate significant resistance compared to wild-type enzyme in identical buffer if long (5 min) extension times were employed. Clone A111 from plate no. 10, and its purified enzyme, reproducibly gave rise to a faster-amplifying signal (Supplementary Figure 1). Subsequent DNA sequencing of clone A111 showed only a single amino acid change, aspartic acid to asparagine at codon 732 (D732N) and one silent mutation G366G. Its plasmid was designated pA111-F7.

### PCR Speed Comparison With Wild-Type Taq Pol

We purified wild-type Taq pol and TaqD732N with the same protocol, and used them at the same concentration, for PCR-amplification of human DNA of variously sized amplicons from the CCR5 gene, all anchored on one side by primer 16.136. We report the PCR products as the bp distance between the 3′-ends of the primers, which ignores the approximate 25–28 bases of opposite primer which must also be copied to complete a PCR cycle. For fastest times, and negligible annealing times, we used 400 nM primers, which is double our normal level of primers. The annealing/extension time at 65°C was varied upward from the minimum machine setting of 1 s per cycle. Although we report the extension times as the thermal cycler machine settings, we estimate that about 3 s of enzyme extension are included in these setting times due to machine hysteresis.

**FIGURE 1:**
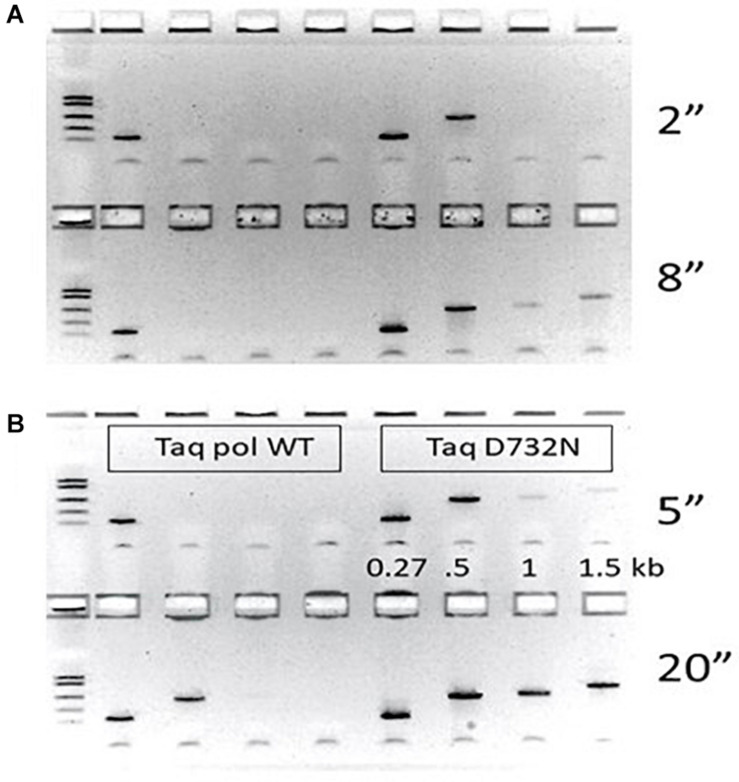
PCR speed tests. PCR performance of wild-type Taq pol on the left half of each agarose gel is compared to TaqD732N on the right half. The gap size (distance between 3′-ends of the primer pairs) was 270, 502, 1000 or 1500 as labeled. Thermal cycler machine settings for annealing/extension time in seconds was tested in pairs with the shorter time on the top half of each gel. **(A)** 2 and 8 s **(B)** 5 and 20 s. Other timings 1, 10, 15, 18, 25, and 30 s are shown in Supplementary Figure 2.

Ignoring faint PCR products (intermediate efficiency), TaqD732N could amplify 502 bp at any setting over 1 s, and 1 kb and 1.5 kb at any setting over 9 s (Figure 1). Wild-type Taq pol required 20 s to amplify 502 bp, and 25–30 s to amplify 1 kb and 1.5 kb (Supplementary Figure 2). Conservatively, we conclude that TaqD732N requires half as much extension time per cycle. The same trend is observable if faint PCR products are considered.

By using actual PCR amplification to measure effective speed, we were able to observe that the effective enzyme speed varies across our test sequences. Both enzymes seemed to be slower for 502 bp (requiring 5 s for TaqD732N, 23 s for wild-type Taq), yet each enzyme needed only another 5 s (10 s for wild-type Taq) to amplify up to 1.5 kb.

### Strand-Displacement by D732N Polymerase Is Effective Enough for LAMP

It has been established that increased processivity (tighter binding) correlates with faster PCR (Wang et al., 2004). We wondered if the increased speed of the D732N mutation might be correlated with tighter binding, and whether the presumed tighter binding might enable observable strand-displacement. If so, our original expectation was that the N-terminal domain (the 5′-flap-endonuclease of full-length Taq pol) would make strand-displacement unobservable, since this nuclease activity should degrade any displaced DNA strand, as indeed it does for the widely used TaqMan assay (Holland et al., 1991). Therefore we constructed Klentaq1 (N-terminal deletion of 278 amino acids deletes the 5′-nuclease) with D732N for our initial strand-displacement tests. Full-length Taq pol and Taq D732N were only included later, as negative controls.

**FIGURE 2:**
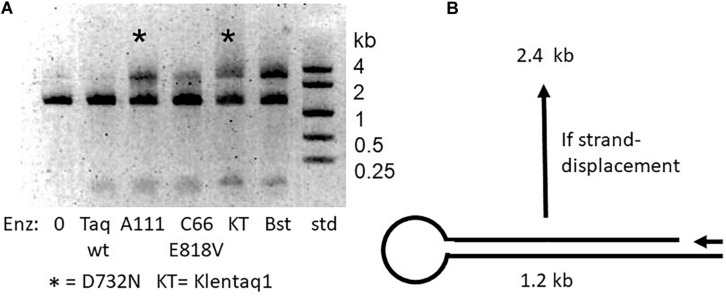
Strand-displacement by mutant enzymes with D732N. **(A)** Half-dumbbell test. Catalyzed DNA synthesis begins at the primer (arrow) and proceeds to and through the loop and back again, only if the polymerase is capable of strand displacement, to make a product 2.4 kb in size. The two D732N mutant versions of Taq (full-length A111 and Klentaq1) are marked with asterisk (*). Bst pol was expected to be able to strand-displace, and was included as a positive control. We explain unextended 1.2 kb template as due to inefficiency of the half-dumbbell construction at unknown point(s) during its preparation. **(B)** Discovery of LAMP ability. A multiply mutant form of Taq pol carrying D732N, named KTflnC4RR (lanes KT*, see text) was tested compared Manta (lanes M), a brand of the large fragment of Bst DNA polymerase (Enzymatics). The banded amplification product which is typical for LAMP appeared at 3 h incubation at 60°C for both enzymes but only for KT* enzyme at 70°C. The middle lane size standards were 1, 2, 4 kb.

As depicted in Figure 2A, we constructed a test DNA template to test for the ability of DNA polymerase to strand-displace 1.2 kb. The 1.2 kb double-strand template was designed to have a gap near one end and a loop at the other end. Successful strand displacement DNA synthesis results in a band at 2.4 kb as the polymerase copies to, around and through the loop, and displaces the non-template DNA strand. Positive control Bst DNA polymerase large fragment, used routinely for LAMP, accomplishes this extension effectively. The D732N mutant versions of full-length Taq pol and Klentaq1 were both discovered to show a significant ability to produce the 2.4 kb product, compared to wild-type Taq pol (and Klentaq1, data not shown).

**FIGURE 3:**
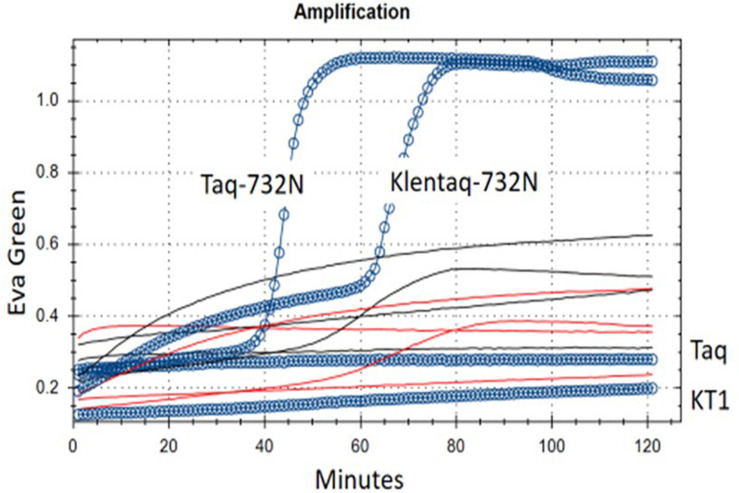
RT-LAMP of MS2 RNA using single-enzyme Taq-D732N or Klentaq1-D732N. Shown are real-time fluorescence traces of reactions with 1/3 μl test enzyme but no RT (reverse transcriptase) enzyme and no Mn^++^, using Eva Green fluorescence as indicator of double-strand DNA amplification. Blue circles/line: complete 26 μl reaction, with 3 ng MS2 RNA. Black lines: MS2 RNA preincubated with RNase I. Red lines, no MS2 RNA added. **Taq and KT1**: Controls wild-type Taq (full-length) and Klentaq1 (N-terminal deletion of 278 a.a.) DNA polymerase. Buffer conditions are 50 mM Tris–HCl pH 8.55, 8 mM ammonium sulfate, 200 μM each dNTP, 0.75 M betaine, 0.025% Brij-58, 68°C. Melt curves for this experiment are shown in Supplementary Figure 2.

We wondered if this strand displacement by D732N mutant Klentaq1 was significant enough to catalyze LAMP. Our first successful LAMP experiment, shown in Figure 2B, used enzyme Klentaq1 with several other mutations in addition to D732N. In total, mutant enzyme KTflnC4RR differs in four ways from wild-type Taq DNA polymerase. (1) The first 278 amino acids are deleted, making it a variant of Klentaq1; (2) D732N; (3) EA742RR; and (4) an insertion, between 738 and 739, of amino acids FLNCCPGCC. Features (3) and (4) were introduced to enhance DNA binding, but in later experiments (RT-PCR) they caused background incorporation. Figure 2B was the initial LAMP discovery experiment, in which KTflnC4RR was found to catalyze LAMP for a lambda DNA target, just as well as Bst large fragment, albeit only after 3 h. This need for this unusually long time was not fully investigated. Further and simplifying experiments, such as Figure 3, showed that the D732N mutation alone is sufficient to confer LAMP ability to Klentaq1.

### Positive Results From the Negative Controls: Strand-Displacement and Reverse Transcriptase Activity

Reverse transcriptases are known to inhibit Taq pol (Sellner et al., 1992; Chandler et al., 1998; Suslov and Steindler, 2005) and PCR, which is why they are routinely heat-denatured after the RT step. We wondered if the inhibition might be due to an interaction with the N-terminal region of Taq pol, and whether the Klentaq1-D732N might be resistant to this inhibitory effect. This question became moot after unexpected results.

When we included full-length Taq pol D732N as the negative (no-RT) control, and also as the negative control for intact N-terminal 5′-exonuclease, we found that it was negative for neither (original experiment with not shown); as demonstrated in Figure 3, Taq-D732N can in fact catalyze RT-LAMP well, and better than Klentaq1-D732N (itself shown here to be a somewhat able RT).

Reverse transcriptase activity is even more surprising than strand-displacement activity; therefore in addition to Klentaq1 and wild-type Taq pol as the no-RT controls, the demonstration in Figure 3. includes two other negative controls against contaminating DNA as the template: (1) no RNA added, and (2) RNase I treatment of the RNA. Both had no amplification of genuine product melting at 86°C (red and black lines of Figure 3 and Supplementary Figure 3A). Another indication of RNA-dependence was that MS2 RNA could not be diluted in water and exposed to 25 or 37°C without rapid loss (decrease of amplicons from RT-LAMP activity; data not shown); this would not happen to carry-over contaminating DNA.

**FIGURE 4:**
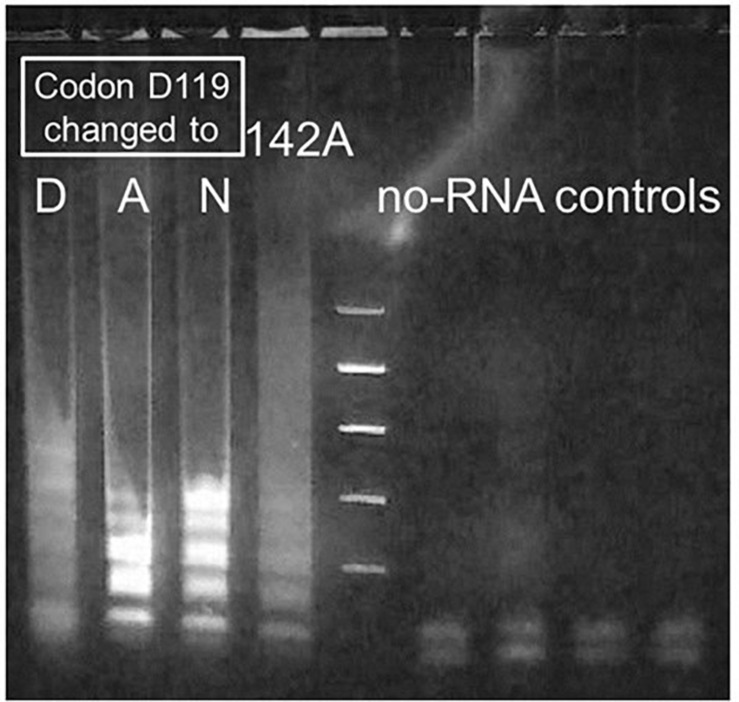
Sharpening of fuzzy bands produced by Taq D732N catalyzing RT-LAMP. MS2 RT-LAMP under conditions as for Figure 3, reacted for only 51 min, catalyzed by four differently mutated enzymes. Codon 732 was D732N and enzyme was full-length Taq pol for all lanes. Lane 1: D119D, i.e., wild-type at codon 119. Lanes 2 and 3: mutations D119A and D119N. Improved clarity of the banding pattern, to make it similar to that observed for standard LAMP, was the result if these mutations near a metal-binding site of the 5′-exonuclease (5′-flap endonuclease) were present in the enzyme. As shown, mutation D142A failed to have this effect.

Agarose gel analysis of similar MS2 RT-LAMP experiments catalyzed by Taq D732N showed a smeared version of the ladder that is usually obtained from LAMP (Figure 4, lane 1). To further verify that the LAMP process is responsible for the amplified DNA, we attempted to disable the 5′-flap endonuclease activity of TaqD732N with a single missense mutation instead of a deletion, to see if the banded pattern appears more sharply, which would be an indication that the 5′-flap endonuclease is responsible for the smeared pattern, and which would not be unexpected. Inspection of the crystal structure 1TAU (Kim et al., 1995) showed a zinc ion in the 5′-flap endonuclease domain where it might be indicating the presence of a magnesium ion required by a nuclease. Two of three mutations tested near this location, both at aspartic acid 119, did indeed allow the banded pattern to survive, as shown in Figure 4, lanes 2 and 3, for D119A and D119N.

**FIGURE 5:**
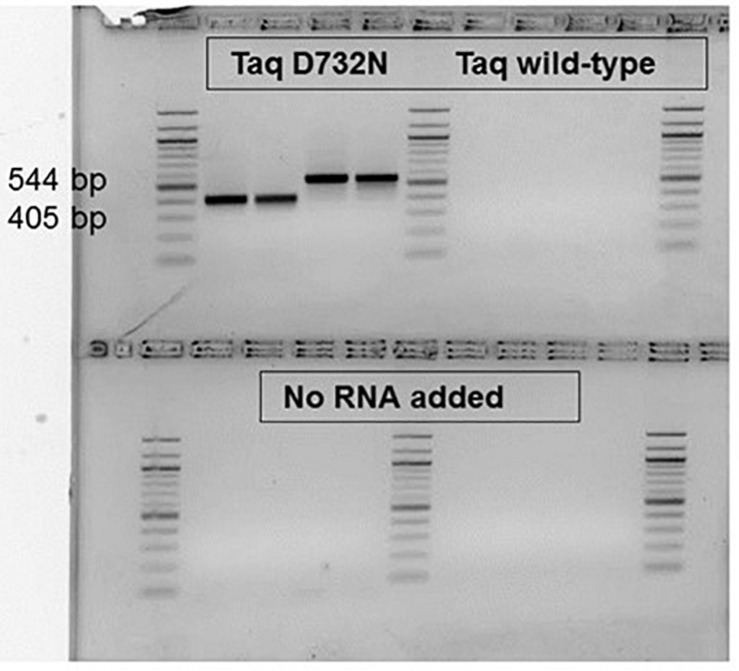
RT-PCR catalyzable by single enzyme Taq D732N but not wild-type Taq. No reverse transcriptase was included, nor was any manganese ion. Two MS2 phage RNA targets, P and K were amplified from 5 pg phage RNA with 0.1 or 0.05 μl D732N mutant or twice as much volume of wild-type Taq (NEB) in 35 μl reactions for 35 PCR cycles. The amplified products were run in a 2.5% agarose gel stained with ethidium bromide, along with a 100 bp DNA ladder (white/black reversed for clarity as printed). As negative control, no RNA was included for the reactions analyzed on the bottom half of the gel. The size of the clearly and successfully amplified bands are 405 and 544 bp (primer-primer gap sizes 359 and 500).

RT-PCR ability, now expected after the RT-LAMP result, was observed for small, ca. 80 bp products (data not shown), and was confirmed for larger products (Figure 5). Proceeding the PCR cycles, 30 min at 68°C was allowed (and was necessary, data not shown) for the RT step, to make the first-strand cDNA. As shown in Figure 5. for product sizes of 405 and 544 bp, the single enzyme TaqD732N is able to catalyze RT-PCR, whilst wild-type Taq cannot, within 35 cycles. The amplification products are shown on an agarose gel, from samples taken after the real-time amplification data and melting point data (Supplementary Figures 3A,B) were collected.

### Reverse Transcription Without Amplification Confirms Large Increase in RT Activity for Taq D732N

To rule out a PCR artifact, we directly compared Taq D732N to wild-type Taq pol for their reverse transcriptase ability. We primed a test RNA template with 4 different primers, and labeled the extension products by incorporation of α-^32^P-dGTP or α-^32^P-dATP. Our intent was to obtain one terminal band from each 25-mer primer, corresponding to the 5′ end of the RNA template, making DNA sizes of 48, 88, 108, and 188 (Supplementary Figure 7A). Overnight exposure of the acrylamide gel to a phosphor screen revealed a much more complicated picture Supplementary Figure 7B (two different primers) and Supplementary Figure 8 (four different primers).

We interpret the many bands at shorter time points as pausing and/or dissociation of the DNA polymerases from the RNA template, largely caused by RNA template structure. Significant secondary structure of this RNA template is suggested by RNA folding software mFold (Zuker, 2003; Supplementary Figure 7C). Although this complexity precludes a simple measure and comparison of DNA polymerase speeds, two conclusions are clearly evident. (1) Wild-type Taq does have some detectible reverse transcriptase activity, showing faint bands corresponding to up to 80 bases of incorporation for one of the primers (17.5′), and (2) Taq D732N is superior at reverse-transcription. At the 30 min time points showing intense bands at the positions expected for terminally extended primers (see asterisks in Supplementary Figure 7B marking 88 nt and 108 nt) there is absolutely no visible band to measure for complete products by wild-type Taq, even when the exposure is adjusted using ImageJ (Supplementary Figure 9). Wild-type Taq pol extension products at this size, if any, are vanishingly low and indistinguishable from background. The time points shown in Supplementary Figure 7B explain why at least 30 min is necessary for the RT step of RT-PCR using TaqD732N under these conditions.

## Discussion

*Thermus aquaticus* DNA polymerase has found wide use for PCR, yet a closely similar enzyme named as from *Bacillus stearothermophilus* (“Bst pol,” used as the N-terminally deleted large fragment) cannot catalyze PCR because it is only thermostable up to about 65°C. Yet near 60°C Bst pol has the useful ability to prime on double-stranded DNA, displacing the DNA strand that was paired to its template (strand-displacement), and thereby to catalyze the sensitive and productive isothermal amplification method known as LAMP (Notomi et al., 2000; Tomita et al., 2008). The analogous N-terminal deletion (-278 amino acids) of Taq pol is known as Klentaq1; both of these N-terminal deletions remove a 5′-exonuclease domain (actually a 5′-flap endonuclease). Neither the full-length Taq pol nor N-terminal deletion of Taq pol can catalyze LAMP. As for RNA assays, an RT (reverse transcriptase) enzyme is routinely combined with these thermostable DNA polymerases to accomplish RT-PCR (reverse transcriptase polymerase chain reaction) or likewise RT-LAMP. The crystal structures of Klentaq1 (Li et al., 1998) and Bst pol (Johnson et al., 2003) are very similar, and their sequences share some 50% identity over the last 422 amino acids which correspond to the polymerase domain.

Family A DNA polymerases are described by crystallographers as resembling a right hand, with fingers and thumb that open and close. Among many crystal structures of Klentaq1, one with primer/template DNA has even been shown to demonstrate both open and closed forms in the same crystal (Li et al., 1998). None of these studies suspect aspartic acid 732 as potentially critical for explaining why Taq pol enzyme cannot perform the functions exhibited by other DNA polymerases, such as copying RNA into DNA (reverse transcriptase) or copying double-stranded DNA (displacing the non-template strand). Such strand displacement is well catalyzed by the homologous large fragment of Bst DNA polymerase when it is used for the isothermal amplification method LAMP (Tomita et al., 2008). Although wild-type Taq can strand-displace to some extent, its 5′-exonuclease (flap endonuclease, FEN) presumably cleaves the displaced strand so much that it is not functional for LAMP. Consistent with the lack of productive strand-displacement by wild-type Taq pol shown in Figure 2A, we could not show any DNA-templated LAMP activity by wild-type Taq pol (Supplementary Figure 5) even under our conditions optimized for RT-LAMP.

Another effective reverse-transcribing variant Taq pol has been described, for an N-terminal deletion, with as few as four mutations (Blatter et al., 2013), and includes a S515R in the thumb near the primer, and M747K near the template but some seven angstroms away from the retained wild-type D732 on the surface of this enzyme. Recently, a thermophilic hot springs phage DNA polymerase of family A has been described that can catalyze RT-LAMP (Chander et al., 2014), and another one that can catalyze RT-PCR (Moser et al., 2012).

We suspect that the added attributes of D732N are not unique among published variants of Taq DNA polymerase. Rather, it is just that these unexpected features have rarely been tested-for in other mutant studies. We suspect that other variants of Taq pol, or other polymerases, may prove to have one or more of these attributes also, with different and/or more amino acid changes. In fact, in control experiments we have observed that large-fragment Bst DNA polymerase has a high and useful level of RT activity, enough that it can carry out RT-LAMP perfectly well in the PCR buffer and relatively low dNTP concentrations that we used in this study (Supplementary Figure 6).

The fact that the wild-type sequence of Taq is so close to being able to catalyze strand-displacement and RT, suggests to us that there may be at least an occasional biological reason for these potential activities. Should tRNA-Asp be mischarged with Asn, or tRNA-Asn be misincorporated at codon 732, the result would be some enzyme made with these activities *in vivo. In vitro*, this could be a source of the low but detectable level of reverse transcriptase activity reported by others for 40 cycles of PCR with wild-type Taq DNA polymerase (Jones and Foulkes, 1989; Shaffer et al., 1990; William and Forget, 1990) and confirmed by us without PCR (this paper). This level of RT activity is so low that it is routine for a “no RT” control to be used for RT-PCR analysis, as a control for contaminating DNA. It is routine to “tune” RT-PCR assays to, i.e., 32–35 cycles, not 40 cycles, since the latter does begin to show the RT activity for wild-type enzyme and short targets (data not shown).

The ability of singly mutated Taq to be faster at PCR and to catalyze LAMP was surprising enough, but for useful reverse transcriptase activity to be a side-effect was not only surprising but also suspicious, since repeated amplification of the same PCR or LAMP product has a well-known potential to contaminate assay workspaces with DNA product (Dieffenbach and Dveksler, 1993). Indeed this began to happen to us, so we implemented improved laboratory hygiene (Dieffenbach and Dveksler, 1993) and included stringent use of DNase I at reaction setup to eliminate possible carry-over, and tested with RNase I to show that the reactions required RNA.

Although mutation D732N appears distant from the primer and template strand in the crystal structures (Supplementary Figure 4), strand-displacement involves three strands, and there is yet no information about the track taken by the displaced strand. The single-strand template takes a sharp right turn near 742,743 in the Bst crystal structure 1L3S (Johnson et al., 2003) and Klentaq1 structure 4XIU (Wu et al., 2015), so the displaced strand must also be in this area, since it was just recently unpaired from the template strand. That could put it near residue 732. Consistent with this reasoning, Taq pol has a positively charged surface RRR (and Bst pol has KQK) in the spatially extrapolated position 715–717 where these positively charged residues could interact with phosphates of the displaced strand, if it is still close to the enzyme. Perhaps the decreased negative charge of mutation D732N is less repelling toward interaction with DNA phosphates.

Improved processivity of Taq pol has been shown to correlate with faster PCR. For instance, this was achieved by the N-terminal addition of 65 amino acids of Sso7d to make Sso7d-Taq (Wang et al., 2004). From this result, and our results of faster PCR, we assume that TaqD732N also has increased processivity. We suspect that Sso7d-Taq has not been tested for RT activity nor LAMP activity, although it has recently been extensively compared to wild-type Taq for PCR (Sundarrajan et al., 2018). It would not surprise us if this or other DNA polymerases that have been modified for increased processivity, or already have high processivity, also have reverse transcriptase and/or strand-displacement activity.

## Conclusion

The enzyme-surface mutation D732N apparently uncovers a reverse transcriptase ability that is inherent to the wild-type active site region of Taq DNA polymerase. Speculatively, if incoming RNA template takes the course proposed for displaced DNA strand, it might interact more closely with 732N than 732D and somehow improve the reverse transcriptase activity to the level observed here, although the presence of the RNA template itself would seem to be a larger perturbation than a single amino acid change. An alternate track for incoming template strand near 732N may also explain the faster PCR speed of TaqD732N, if this location binds to the template DNA more tightly, causing increased processivity. Productive strand-displacement, rather than the wild-type enzyme’s concomitant flap endonuclease/exonuclease degradation of the displaced strand, has also resulted from the D732N mutation. Use of TaqD732N DNA polymerase should allow an increased range of temperatures and protocols to be employed for RT-LAMP and RT-PCR assays. An additional mutation at D119, designed to decrease 5′-exonuclease activity, showed further-enhanced LAMP activity.

## Data Availability Statement

The original contributions presented in the study are included in the article/Supplementary Material, further inquiries can be directed to the corresponding author.

## Author Contributions

WB discovered the increased speed of TaqD732N, its LAMP and RT-LAMP abilities, and wrote the manuscript. MK designed the screen for mutant enzymes and found D732N. ZZ discovered the RT-PCR ability. All authors contributed to the article and approved the submitted version.

## Conflict of Interest

The authors have applied for a patent including these discoveries, and are associated with the for profit/loss company DNA Polymerase Technology, Inc.
