# Supplementary material for: A Single Amino Acid Change to Taq DNA Polymerase Enables Faster PCR, Reverse Transcription and Strand-Displacement
Source: Front Bioeng Biotechnol. 2021 Jan 14;8:553474. doi: 10.3389/fbioe.2020.553474 (PMC7841393; doi:10.3389/fbioe.2020.553474)
Supplement: Supplementary file 1 [file Data_Sheet_1.PDF]

A single amino acid change to Taq DNA polymerase enables faster PCR, reverse transcription and strand-displacement

Wayne M Barnes[1,2]*, Zhian Zhang[2] , Milko B Kermekchiev[2]

[1] Biochemistry and Molecular Biophysics, Washington University School of Medicine, St. Louis, Missouri, USA
[2] DNA Polymerase Technology, Inc., St. Louis, Missouri, USA
Correspondence should be addressed to WMB. (wayne@barnes1.wustl.edu)

SUPPLEMENTARY MATERIALS

Table of Contents

Supplementary Text …………………………………………..3

Supplementary Figures 1 – 6 ………………………………….4

Supplementary References …………………………………..13

Supplementary Text

TaqD732N does not produce a normal distinct ladder of gel bands during LAMP, as does Bst pol and KlentaqD732N, probably because its 5'-flap-exonuclease (which we had previously expected to prevent LAMP altogether) does have a degrading effect on the band resolution. There is a patented mutation G46D[1] that is described to reduce the 5'-exonuclease activity and could be expected to restore the ladder of bands that is typical for LAMP. Despite several attempts, we were unable to introduce this mutation into our expression plasmid, with or without D732N. Perhaps this mutation is lethal to either our host bacteria or to the replication of the expression plasmid vector pUC19, when expressed in this host. Our mutations at codon D119 were easy to introduce, and did successfully restore the banded pattern to RT-LAMP products, consistent with effective reduction or elimination of the 5' nuclease activity (**Fig. 4**).

Repetition information

Fig. 3. This RT-LAMP experiment was performed twice. The first time, the RNase I treatment was on ice for 5 minutes, and it showed less effect for suppression of the Taq-732N amplification. Also the first time, the Klentaq-732N failed completely, perhaps because the primers were not added successfully, or they fell into the DNase prematurely. Klentaq-732N worked well 4/4 reactions previously, while testing the DNase treatment protocol.

At the low amounts used, RNAse I treatment was ineffective in full-strength reaction buffer, which is why our treatment was in 1/10 strength buffer, and why the RNase treatment time is effectively ended by addition of the full reaction mix. If the MS2 RNA was suspended in water (zero-strength buffer), it degraded in minutes at room temperature, as mentioned in the text.

DNA sequencing of the ORF of plasmid A111-F7 included several repetitions during the construction of the 5'-endonuclease mutants shown in Figure 4, since these mutations, which used the plasmid pA111-F7 as the parent, were also fully sequenced.

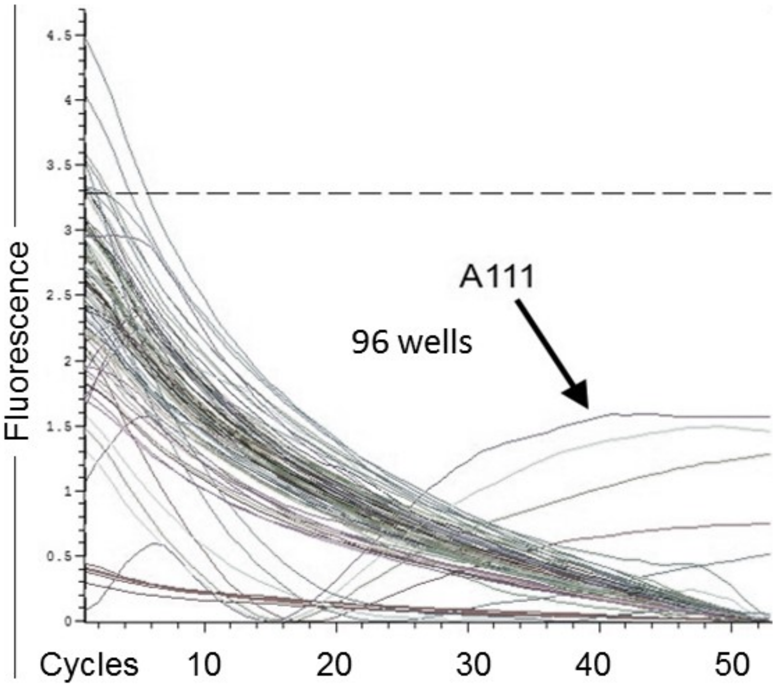

Supplementary Figure 1. Mutagenized library activity screen using crude E.coli extract as PCR enzyme and template. Raw data is charted for 96 PCR reactions indicated by SyBr Green fluorescence. The highest rising curve, displaying lowest Ct value, proved to be Taq DNA polymerase clone A111, with a single mutation D732N.

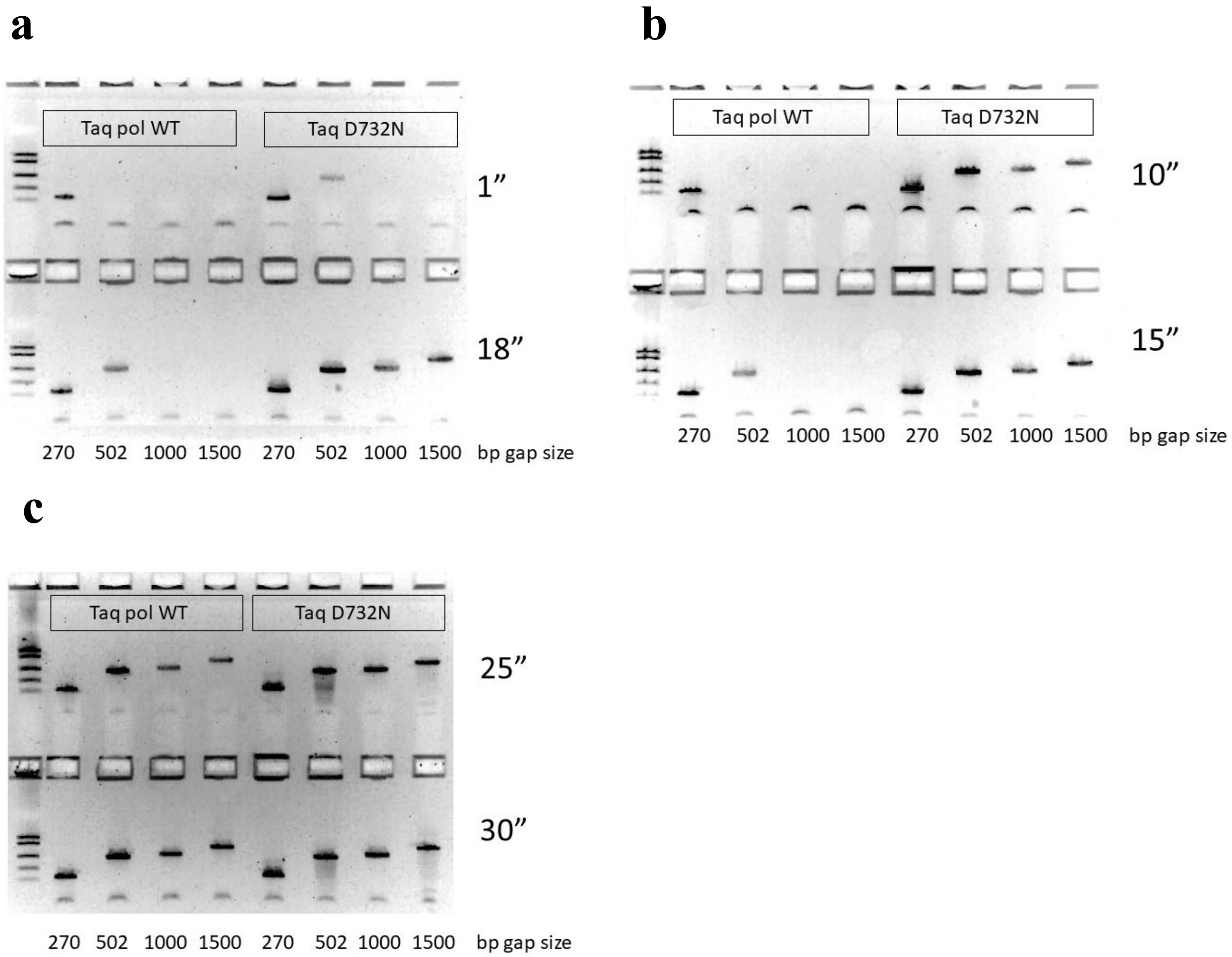

Supplementary Figure 2. Additional time points (anneal/extend times at 65 degrees C.) for PCR speed tests, (**a**) 1 sec. and 18 sec. (**b**) 10 sec. and 15 sec. (**c**) 25 sec. and 30 sec. Size standards are 4, 2, 1, 0.5, 0.25 kb.

a

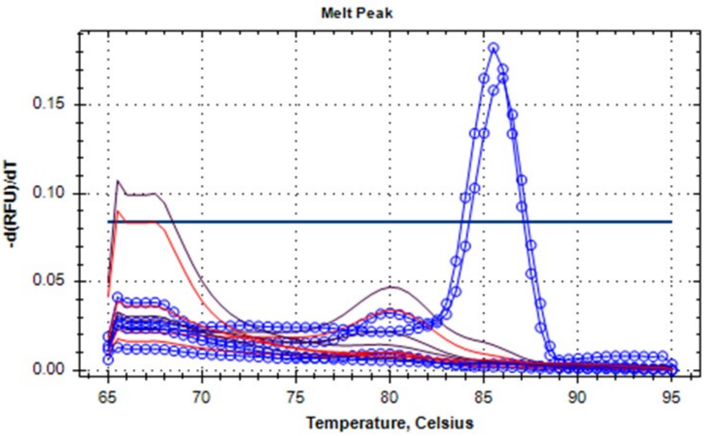

Supplementary Figure 3. Fluorescence data. (a) Melt curves for the RT-LAMP real-time reactions of Figure 3. Legitimate product catalyzed by Taq732N and Klentaq732N melts at 85-86°. No products from non-template and RNAse-treated template, nor wild-type Taq and Klentaq1, melted in this range. **(b)** SYBR green real-time fluorescence for RT-PCR of **Fig. 5.** The fastest 4 curves are from the TaqD732N reactions. **(c)** Melt curves for this experiment. The 4 species melting at 87 C are the TaqD732N products.

b

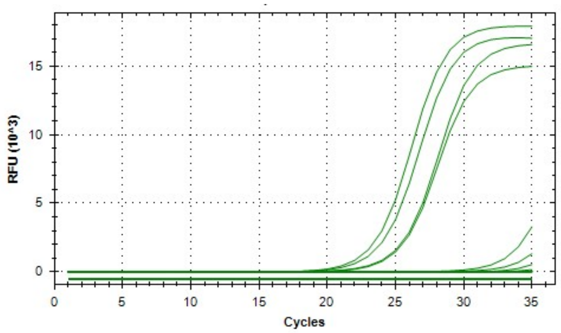

c

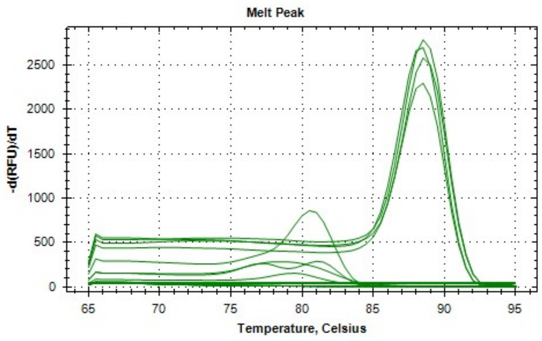

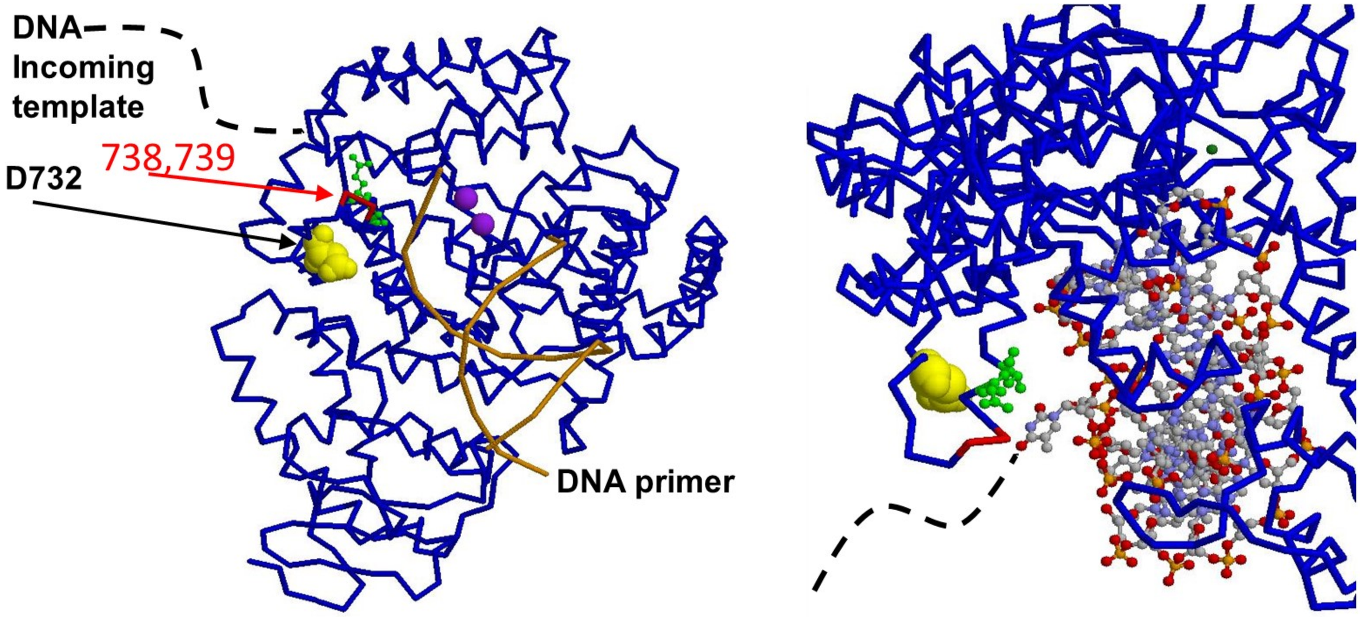

a

b

Supplementary Figure 4 Rasmol (2.7.2 Roger Sayle) renditions of crystal structures of Klentaq1. (**a**) 3KTQ.pdb with DNA primer and template. Incoming template is drawn with a dashed line to show its approximate position. This part of the template structure was not determined, since 3 bases in this area were disordered, and more bases of template were not even included in the crystallization. Crystallization for a structural study including a displaced DNA strand or RNA template has not yet been attempted. A few amino acids mentioned in the text are highlighted: the position of mutation D732N is indicated as yellow space-filled; amino acids 742 and 743 are colored green, and positions 738 and 739 are shown in red as C-alpha of the protein backbone. The active site is approximately located by the 2 magnesium ions drawn as purple (A) or green (B) spheres. DNA primer and template are shown as orange backbone.
(b) Klentaq1 crystal structure 4XIU from another angle. Almost same coloring, except the DNA is shown as CPK-colored atoms. This structure has determined one more ordered base of the template, a 5'-T, showing entrance to the enzyme near residues 739 and 742, but still not near 732.

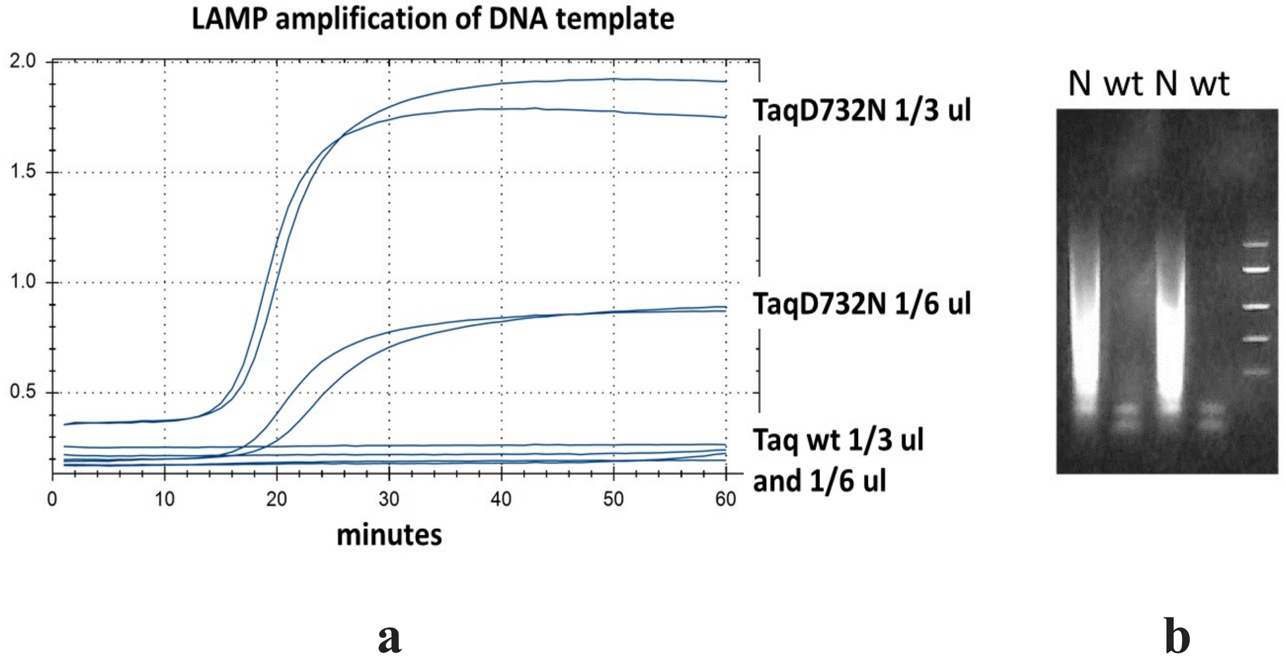

Supplementary Figure 5. Taq pol wild-type cannot catalyze LAMP under optimized conditions. Once we realized that Taq D732N has reverse transcriptase activity and can catalyze RT-LAMP, we dispensed with a classical RT step for further optimizations to arrive at a RT-LAMP buffer composition. To rule out the possibility that wild-type Taq pol can catalyze LAMP from DNA template under our more optimal conditions, we first generated a test DNA template by RT-PCR (30 cycles) catalyzed by TaqD732N,D119A using 1 ng of MS2 RNA template in a reaction volume of 50 ul, followed by PEG+SDS purification. Outside-nesting PCR primers were (MS2PL: ATAGAGAAGG TTTCTTACA TGACAAAT and MS2PR': TCTGAT CCACGGCGCAC ATTGGTCTCGGA). DNAse I and UDG were not used to set up the 25 ul LAMP reactions, which were seeded with ca. 5 ng of the purified RT-PCR DNA product. Otherwise this LAMP used the same primers and buffer as described for MS2 RT-LAMP, incubated at 68 deg. C. for 60 minutes. **a)** Eva Green fluorescent traces catalysed by 1/3 ul or 1/6 ul enzyme per 25 ul reaction, as indicated. **b)** Agarose gel positive control lanes **N** (TaqD732N) analyse reactions catalysed by 1/3 ul enzyme per 25 ul reaction. Lanes **wt** tested wild-type Taq pol made by us and used at same concentration. One third ul of enzyme is 10 times more than would be necessary for PCR.

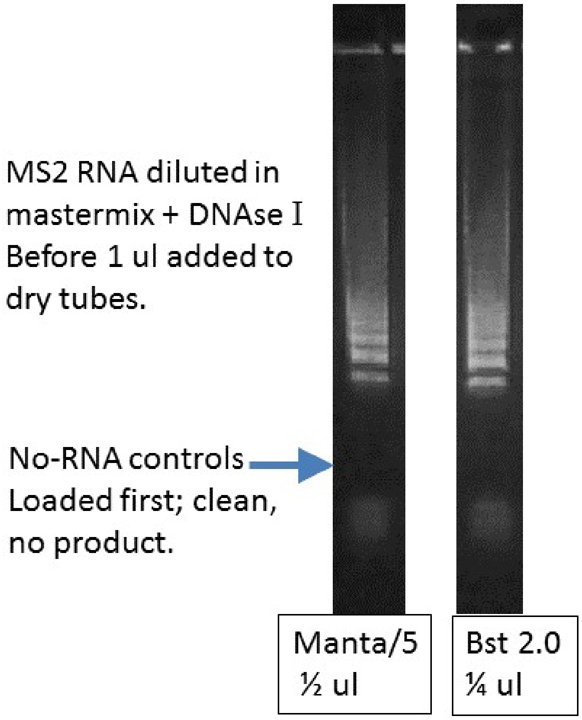

Supplementary Figure 6 Bst DNA polymerase can, as the single enzyme, catalyze RT-LAMP using PCR buffer and low dNTPs. No-RNA controls were loaded first in the same lanes, and were observed to show no product (Lonza computer camera gel system), before loading the +RNA reactions. MS2 RNA reactions were loaded second, and show the banded pattern typical for LAMP products. No RT enzyme nor Mn ions were added. Enzymatics Bst enzyme (Manta) was diluted 5-fold before addition of final ½ ul per 26 ul reaction. NE Biolabs enzyme (Bst 2.0) was added without dilution at final ¼ ul per reaction. Reactions were incubated at 65° C. for 51 minutes.

```
                           <---Primer 17.2'      <---Primer 17.3' 43 to go
  1 GGGAGACCAC AACGGUUUCC CUCUAGAAAU AAUUUUGUUU AACUUUAAGA AGGAGAUAUC

                           <---Primer 17.4' 83 to go

 61 CAUGGCUAAG AUGGCUUUCA CCCUGGCUGA CCGUGUUACC GAAGAAAUGC UGGCGGACAA

                                                 <---Primer 17.5' 163 to go

121 AGCUGCGCUG GUCGUUGAAG UUGUCGAAGA GAACUAUCAC GACGCGCCAA UUGUCGGUAU

181 CGCAGUAGUU AACGAGCACG GCCGUUUCUU CCUGCGCCCU GAGACAGCAC UGGCCGACCC

241 GCAGUUCGUU GCUUGGCUGG GUGACGAAAC UAAAAAAAAG UCUAUGUUCG AUUCCAAACG

301 UGCAGCAGUA GCGCUGAAAU GGAAAGGCAU CGAGCUCAGC GGCGUUUCCU UCGACCUGCU

361 GCUUGCGGCC UACCUGCUGG AUCCAGCUCA GGG
```

Supplementary Figure 7A. RNA sequence of test template used for simple extension reverse transcriptase assay. The **<** position is the 3’ end of each primer. All of these primers are 25 nt in length. “to go” refers to the distance to the expected 5’-end of the T7 RNA polymerase transcript, a position only and partially achieved by Taq D732N enzyme in the 20 and 30 minute time points for primer 17.4’ (108 nt product), and primer 17.5’ (188 nt product), both as doublets.

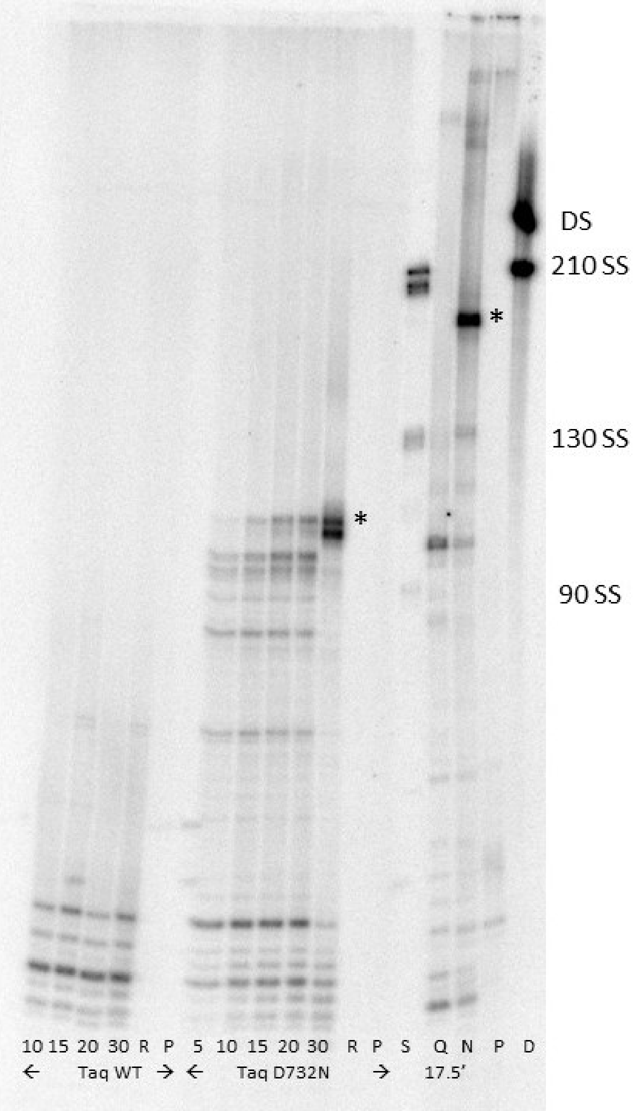

Supplementary Figure 7B. Simple extension reverse transcriptase assay. Radioactive label was α^{32}P-dGTP, with all 4 dNTP at 200 uM each. Lanes marked 5,10,15,20,30 refer to minutes at 68° C. Lanes "Taq WT" and Q (30 min. extension) demark wild-type Taq pol enzyme reactions. Lanes "Taq D732N" and N (30 min. extension) demark mutant enzyme reactions. Band positions marked SS refer to bands of single-stranded DNA in lane S. Band DS is partially denatured PCR product of size 210 bp in lane D and apparently contains both single-stranded and double-stranded DNA. Lanes R (RNA-only) and P (primer-only) are controls. Two asterisks (*) demark bands that we identify as fully-extended DNA. Except for 3 lanes Q N P marked 17.5', lanes were primed with primer 17.4' (see Supplementary Figure 7A).

Supplementary Figure 7C

Possible secondary structure of the first 188 nucleotides of the RNA template, output by software mFold [2]. The approximate position of primers 17.5' (left) and 17.4' (right) are shown as arrows. The actual template RNA structure after a primer has annealed could be much different.

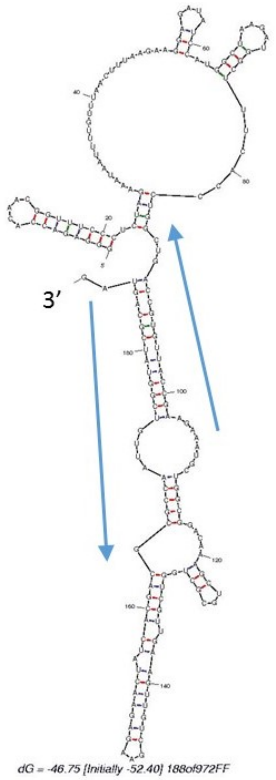

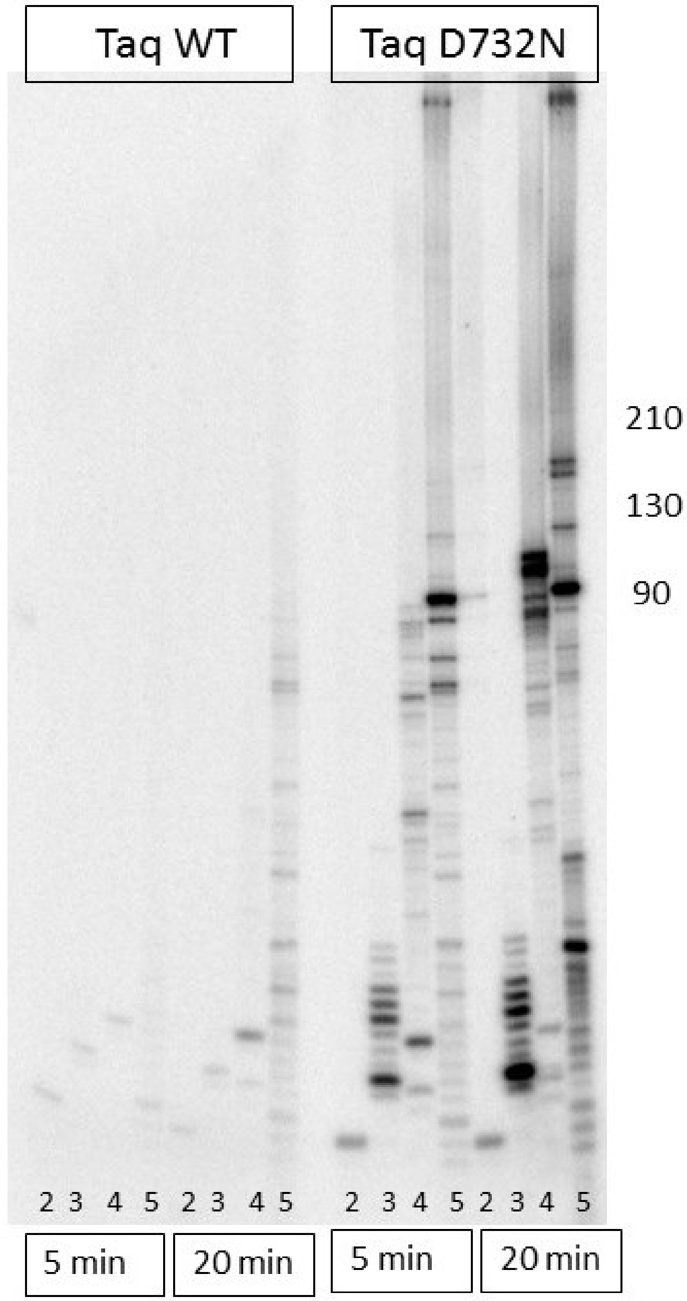

Supplementary Figure 8 Reverse transcriptase demonstration comparing wild-type Taq pol to Taq D732N, simple primer extension on RNA template without temperature cycling. Four primers were used, positioned as in Figure 7A. Single-stranded DNA sizes are approximate, by comparison to size standards in Supplementary Figure 7B. Radioactive label was α^{32}P-dATP, with 80 uM dATP and 200 uM each dGTP, dTTP and dCTP. We identify the uppermost bands near the top of the gel, possibly, as folded DNA strands. Lanes labelled 2, 3, 4, or 5 refer to priming by primers 17.2’, 17.3’, 17.4’, and 17.5’.

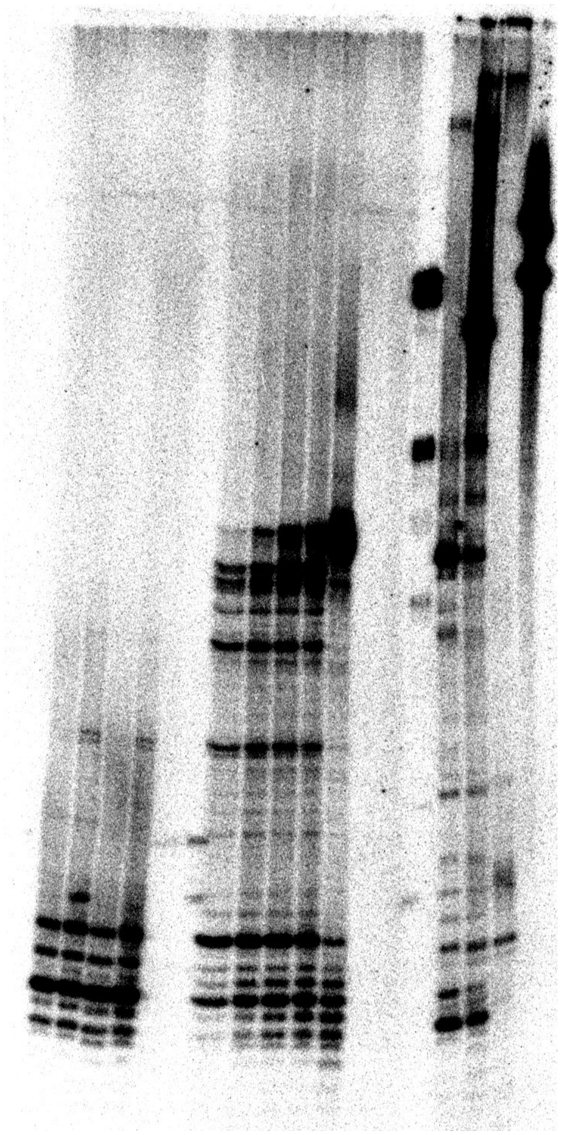

Supplementary Figure 9. Simulated overexposure created by using the Image/Adjust/Brightness/Contrast feature of ImageJ [3]. Note the complete lack of product by WT Taq at the positions labelled with an asterisk (*) in **Supplementary Figure 7B.**

--

Supplementary References

1. Abramson, R.D. & Gelfand, D.H. 5' to 3' exonuclease mutations of thermostable DNA polymerases. US Patent 5,466,591 (1995).
2. Zuker, M. Mfold web server for nucleic acid folding and hybridization prediction. *Nucleic acids research* **31**, 3406-3415 (2003).
3. Schneider, C.A., Rasband, W.S. & Eliceiri, K.W. NIH Image to ImageJ: 25 years of image analysis. *Nature methods* **9**, 671 (2012).
